# Peptidylarginine deiminase 4 concentration, but not *PADI4* polymorphisms, is associated with ICU mortality in septic shock patients

**DOI:** 10.1111/jcmm.13717

**Published:** 2018-07-25

**Authors:** Nara Aline Costa, Ana Lúcia Gut, Paula Schmidt Azevedo, Bertha Furlan Polegato, Eloá Siqueira Magalhães, Larissa Lumi Watanabe Ishikawa, Rita de Cassia Siqueira Bruder, Evelyn Aparecida da Silva, Renan Braga Gonçalves, Suzana Erico Tanni, Marcelo Macedo Rogero, Marina Maintinguer Norde, Natália Baraldi Cunha, Leonardo Antonio Mamede Zornoff, Sergio Alberto Rupp de Paiva, Marcos Ferreira Minicucci

**Affiliations:** ^1^ Department of Internal Medicine Botucatu Medical School UNESP – Univ Estadual Paulista Botucatu Brazil; ^2^ Department of Microbiology and Immunology Institute of Biosciences of Botucatu UNESP ‐ Univ. Estadual Paulista Botucatu Brazil; ^3^ Nutrition Department School of Public Health University of São Paulo São Paulo Brazil

**Keywords:** citrullination, neutrophils extracellular traps, peptidylarginine deiminase 4, sepsis, single nucleotide polymorphisms

## Abstract

The objective of our study was to evaluate the association between peptidylarginine deiminase 4 (PAD4) concentration and its polymorphisms with mortality in patients with septic shock**.** We prospectively evaluated 175 patients aged over 18 years with septic shock upon intensive care unit (ICU) admission. However, 48 patients were excluded. Thus, 127 patients were enrolled in the study. At the time of the patients’ enrollment, demographic information was recorded. Blood samples were taken within the first 24 hours of the patient's admission to determine serum PAD4 concentrations and its polymorphism *PADI4*_89 [*rs*11203366], *PADI4*_94 [*rs*2240340] and *PADI4*_104 [*rs*1748033]. The mean age was 63.3 ± 15.2 years, 56.7% were male, PAD4 concentration was 4.62 (2.48‐6.20) ng/mL and the ICU mortality rate was 67.7%. The patients who died in the ICU had higher APACHE II and Sequential Organ Failure Assessment (SOFA) scores. In addition, PAD4 concentration was higher in patients who died during ICU stay. However, there were no differences regarding *PADI4* polymorphisms and ICU mortality. In the logistic regression models, PAD4 concentrations were associated with ICU mortality when adjusted for APACHE II score and lactate (OR: 1.477; CI 95%: 1.186‐1.839; *P* < .001), and when adjusted for age, gender and APACHE II score (OR: 1.392; CI 95%: 1.145‐1.692; *P* < .001). In conclusion, PAD4 concentration, but not *PADI4*_89, *PADI4*_94 and *PADI4*_104 polymorphisms, is associated with ICU mortality in septic shock patients.

## 1 INTRODUCTION

Sepsis and septic shock are considered major public health problems and the leading causes of mortality in intensive care unit (ICU) patients.^1^ Nowadays, sepsis is characterized as a life‐threatening organ dysfunction caused by a dysregulated host response to infection, in which both pro‐inflammatory and anti‐inflammatory mechanisms contribute to clearance of infection and tissue recovery on the one hand and organ injury and secondary infections on the other.^1^ Thus, the study of pathways that modulate the inflammatory process could improve the current knowledge in sepsis physiopathology and identify potential therapeutic targets. In this scenario, the citrullination process has become a hot topic, and has been identified in several inflammatory diseases.^2^, ^3^

Citrullination represents the calcium‐dependent conversion of peptidylarginine to peptidylcitrulline, which is catalyzed by peptidylarginine deiminase (PAD) enzymes. This enzymatic conversion has an important role in immune system function and in gene regulation.^2^, ^3^ In humans, there are five highly related calcium‐dependent PADs, which have been designated as PADs 1‐4 and PAD6. Among PAD isoforms, PAD4 is extremely important because it is present in different tissues, and it is the only PAD that is present in cell's nucleus. PAD4 consists of 663 amino acid residues with 74 kDa molecular weight. PAD4 participates in post‐translational modifications of histones, one of the major focus areas of epigenetic research.^2^, ^4^ Citrullination of histones, in particular histone H3, is one of the major points for inflammatory signals that trigger the neutrophil response to infections.^2^, ^3^, ^4^, ^5^ Nevertheless, citrullinated histone H3 is also important in the development of neutrophils extracellular traps (NETs).^5^, ^6^ It has been reported that elevated levels of NETs in the plasma may predict multiple organ dysfunction and sepsis in trauma patients.^6^

The role of PAD in sepsis has been evaluated in some experimental studies. In these PAD4 inhibition was associated with improved survival.^5^, ^7^, ^8^ Despite the importance of PAD4 in sepsis, the association between PAD4 concentration and mortality has not yet been evaluated in patients with septic shock. In addition, some polymorphisms have been found in human *PADI4* gene. The *PADI4* gene is located on the short arm of chromosome 1 at position 36.13 and functional polymorphisms, such as *rs*11203366, *rs*2240340 and *rs*1748033 have been associated with susceptibility to rheumatoid arthritis in different populations. However, the majority of these studies evaluated the relationship between *PADI4* gene polymorphisms with rheumatoid arthritis development.^9^, ^10^

Thus, the aim of our study was to evaluate the association between PAD4 concentration and its polymorphisms with mortality in patients with septic shock.

## 2 MATERIALS AND METHODS

This prospective observational study is a subanalysis of a larger unpublished study, which evaluated the relationship between oxidative stress and mortality in patients with septic shock. The study was conducted from May 2014 to June 2015 in patients admitted to the ICU of our hospital. The protocol was approved by the Ethics Committee of our institution (30457414.7.0000.5411). Written informed consent was obtained from all patients or relatives prior to their inclusion in the study.

Patients were eligible for enrollment if they were 18 years or older and had septic shock on ICU admission. Exclusion criteria were a delay in septic shock diagnosis (longer than 24 hours), pregnant women, patients with confirmed brain death and patients in palliative care and use of vasoactive drugs for <24 hours.

Upon admission, patient demographic information, the Acute Physiology and Chronic Health Evaluation (APACHE II) score and the Sequential Organ Failure Assessment (SOFA) score were recorded. Blood samples were taken within the first 24 hours of the patient's admission to determine serum peptidylarginine deiminase 4 and its polymorphism *PADI4*_89 [*rs*11203366], *PADI4*_94 [*rs*2240340] and *PADI4*_104 [*rs*1748033]. The ICU mortality rate was recorded. Septic shock was defined according to the Surviving Sepsis guidelines.^11^

### 2.1 Laboratory analysis

A hemogram was performed with a Coulter STKS hematological auto analyzer. Total serum levels of C‐reactive protein (CRP), albumin, creatinine and urea were measured performed with the dry chemistry method (Ortho‐Clinical Diagnostics VITROS 950^®^, Johnson & Johnson). Lactate was measured performed with the Roche OMNI S™ Blood Gas Analyzer (Roche Diagnostics, Basel, Switzerland).

### 2.2 PADI4 gene polymorphism

DNA was isolated from frozen blood samples performed with a method described elsewhere. DNA integrity was checked using a 1% agarose gel, whereas DNA concentration was measured performed with a Nanodrop 8000 spectrophotometer (Thermo Scientific, Waltham, MA, USA). The genotyping of the polymorphisms of the PAD4 gene *rs*11203366, *rs*2240340 and *rs*1748033 was performed by the TaqMan Open Array (Applied Biosystem, Foster City, CA, USA), following the manufacturer's instructions.^12^

### 2.3 Serum PAD4 concentration

The serum levels of PAD4 were assessed using enzyme linked immunosorbent assay (ELISA), according to the manufacturer's instructions (Cloud‐Clone Corp, Houston, TX, USA). The sensitivity of the assay was 0.137 ng/mL.

### 2.4 Statistical analysis

Data are expressed as the mean ± SD, the median (including the lower and upper quartiles) or percentage. Hardy‐Weinberg equilibrium was determined performed with Fisher's Exact test, and haplotype frequency, haplotype crude association with mortality, and linkage disequilibrium were calculated performed with Haploview software version 4.2 (Broad Institute, Boston, MA, USA). Statistical comparisons between two groups for continuous variables were performed performed with Student's *t* test for parameters with a normal distribution. If data were not normally distributed, comparisons between two groups were made performed with the Mann‐Whitney *U*‐test. Comparisons between three groups with non‐normal distribution were performed with Kruskal‐Wallis test. Fisher's test or the Chi‐Square test was used for all categorical data. Logistic regression was used to evaluate the association between PAD4 concentration and ICU mortality. We constructed two regression models. PAD4 concentration was tested as a continuous variable. In the first model, PAD4 concentration was adjusted with parameters that exhibited significant differences in the univariate analysis. The only exceptions were variables with high collinearity among them (SOFA score). In the other, PAD4 was adjusted for age, gender, and APACHE II score. Data analysis was performed performed with SigmaPlot software for Windows v12.0 (Systat Software Inc., San Jose, CA, USA). The significance level was 5%.

## 3 RESULTS

**Figure 1:**
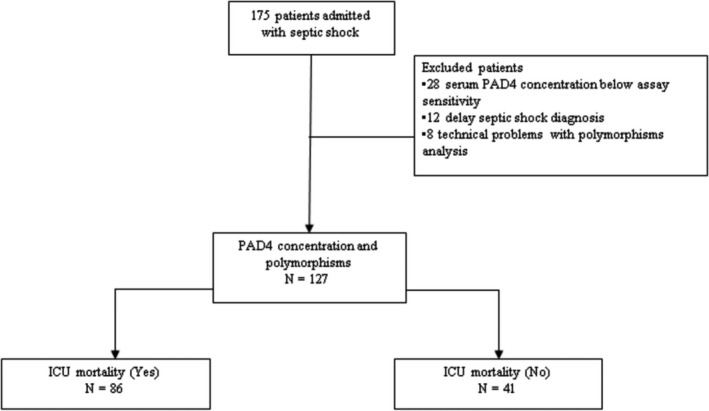
Flow diagram of studied patients with septic shock

During the study, 175 consecutive patients were admitted to the ICU with the diagnosis of septic shock; however, 12 patients were excluded because of delayed diagnosis of septic shock, 28 because of serum PAD4 concentration below assay sensitivity and 8 because of technical problems with polymorphism analysis. Thus, we evaluated 127 patients (Figure 1). The mean age was 63.3 ± 15.2 years, 56.7% were male, PAD4 concentration was 4.62 (2.48‐6.20) ng/mL and the ICU mortality rate was 67.7%.

The demographic and laboratory data are presented in Tables 1 and 2. Patients who died in the ICU had higher APACHE II and SOFA scores. In addition, PAD4 concentration was higher in patients who died during ICU stay. However, there were no differences regarding *PADI4* polymorphisms. In relation to laboratory data, lactate levels were higher in patients who died during ICU stay.

**Table 1:** Demographic and *PADI4* polymorphisms data of 127 patients with septic shock

Variables	ICU mortality		*P* value
	Yes (n = 86)	No (n = 41)	
Age, (years)	66.0 (58.0‐73.0)	63.0 (50.0‐73.5)	.337
Male, n (%)	45 (52.3)	27 (65.8)	.212
APACHE II score, n (%)	19.7 ± 6.6	15.9 ± 6.7	.003
SOFA score, n (%)	10.0 (8.7‐12.0)	8.0 (7.0‐10.5)	<.001
Sepsis focus, n° (%)			
Respiratory	55 (64.0)	18 (44.0)	.221
Abdominal	16 (18.6)	13 (31.7)	
Urinary	5 (5.8)	5 (12.2)	
Others	10 (11.6)	5 (12.1)	
*PADI4* (rs11203366), n (%)			
GG	18 (21.0)	6 (14.6)	.642
AG	36 (41.8)	20 (48.8)	
AA	32 (37.2)	15 (36.6)	
*PADI4* (rs2240340), n (%)			
TT	18 (21.0)	4 (9.8)	.219
CT	37 (43.0)	23 (56.1)	
CC	31 (36.0)	14 (34.1)	
*PADI4* (rs1748033), n (%)			
TT	10 (11.6)	3 (7.3)	.697
CT	37 (43.0)	20 (48.8)	
CC	39 (45.4)	18 (43.9)	

APACHE II, Acute Physiology and Chronic Health Evaluation; ICU: intensive care unit; PAD 4: peptidylarginine deiminase 4; SOFA: Sequential Organ Failure Assessment.

Data are expressed as the mean ± SD median (including the lower and upper quartiles) or percentage.

**Table 2:** Laboratory data of 127 patients with septic shock

Variable	ICU mortality		*P* value
	Yes (n = 86)	No (n = 41)	
PAD4, (ng/mL)	4.9 (3.1‐6.6)	3.5 (1.3‐5.5)	.001
Lactate, (mmol/L)	2.6 (1.5‐3.9)	1.4 (1.0‐2.7)	.004
Hemoglobin, (g/dL)	11.0 ± 2.0	11.4 ± 1.8	.202
Hematocrit, (%)	32.7 ± 6.3	33.8 ± 5.3	.339
Leucocytes, (10^3^/mm^3^)	16.8 (12.8‐23.7)	16.4 (12.1‐23.0)	.477
CRP, (mg/dL)	33.5 (22.6‐43.3)	34.5 (23.3‐44.8)	.799
Albumin, (g/dL)	2.2 (1.9‐2.5)	2.3 (1.8‐2.6)	.632
Urea, (mg/dL)	102 (64‐159)	81 (47‐149)	.163
Creatinine, (mg/dL)	1.9 (0.8‐3.4)	1.5 (0.7‐2.9)	.331

CRP, C‐reactive protein; ICU, intensive care unit; PAD 4, peptidylarginine deiminase 4.

Data are expressed as median (including the lower and upper quartiles).

**Figure 2:**
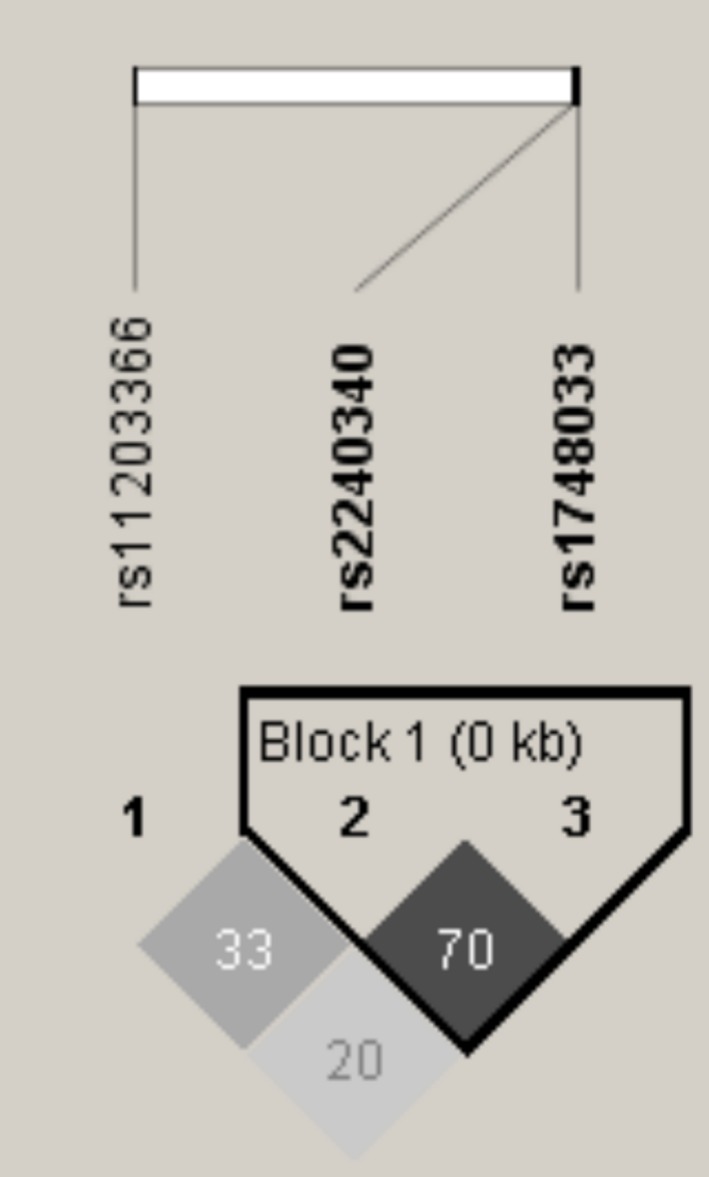
Haploview plot of *PADI4* gene SNP. Within each square is presented the pairwise correlation coefficient (*r* ^2^) LD plots white, (*r* ^2^ = 0), shades of grey, (0 < *r* ^2^ < 1), black, (*r* ^2^ = 1), and “rs” in bold refer to haplotype block

The genotype frequencies for the *rs*11203366 polymorphism were 18.9% for GG, 44.1% for AG and 37.0% for AA; for the *rs*2240340 polymorphisms they were 17.3% for TT, 47.3% for CT and 35.4% for CC; and for the *rs*1748033 polymorphism they were 10.2% for TT, 44.9% for CT and 44.9% for CC. These frequencies are consistent with those expected under the Hardy‐Weinberg equilibrium. SNP *rs2240340* and rs*1748033* formed a haplotype block (Figure 2). In addition, haplotype had no statistically significant association with ICU mortality (data not shown), and PAD4 concentrations were not different between polymorphism genotypes. (Table 3).

**Table 3:** Association between PAD 4 concentration and its polymorphisms in 127 patients with septic shock

Variables	PAD 4 concentration (ng/mL)		*P* value
*PADI4* (rs11203366), n (%)	n		
GG	24	3.67 (2.23‐5.86)	.182
AG	56	4.43 (2.10‐6.20)	
AA	47	5.45 (3.43‐6.52)	
*PADI4* (rs2240340), n (%)			
TT	22	5.10 (3.18‐6.64)	.193
CT	60	3.68 (1.93‐6.20)	
CC	45	4.65 (2.16‐5.87)	
*PADI4* (rs1748033), n (%)			
TT	13	4.56 (3.05‐6.50)	.752
CT	57	4.28 (2.32‐6.39)	
CC	57	4.64 (2.33‐5.87)	

PAD 4, peptidylarginine deiminase 4.

In the logistic regression models, PAD4 concentrations were associated with ICU mortality when adjusted for APACHE II score and lactate (OR: 1.477; CI 95%: 1.186‐1.839; *P* < .001), and when adjusted for age, gender and APACHE II score (OR: 1.392; CI 95%: 1.145‐1.692; *P* < .001). (Table 4).

**Table 4:** Logistic regression model for the prediction of ICU mortality in 127 patients with septic shock

Variable	OR	CI 95%	*P* value
PAD4 (ng/mL)^a^	1.320	1.111‐1.569	.002
PAD4 (ng/mL)^b^	1.477	1.186‐1.839	<.001
PAD4 (ng/mL)^c^	1.392	1.145‐1.692	<.001

^a^Unadjusted; ^b^Adjusted by APACHE II score and lactate; ^c^Adjusted by gender age and APACHE II score.

OR: Odds ratio; CI95%: confidence interval 95%.

It is interesting to observe that the results were the same even when we included the patients with serum PAD4 concentration below assay sensitivity, and set the value with the detection limit of the kit. (Data not shown).

## 4 DISCUSSION

The objective of our study was to evaluate the association between PAD4 concentration and its polymorphisms with mortality in patients with septic shock. This study showed that PAD4 concentration was associated with ICU mortality in septic shock patients, independent of polymorphism genotype.

Septic shock is the major cause of death in ICU, and its incidence is increasing, reflecting aging populations and greater recognition.^1^ In our study, 67.7% of the patients died during ICU stay. Although this is a very high mortality rate, it is in accordance with the septic shock mortality observed in other studies in Latin America.^13^, ^14^ As we already expected, the patients who died during ICU stay were more severely ill and had more organ dysfunction compared to those who survived.

In this scenario, a better understanding of sepsis physiopathology is important to identify new therapeutic targets. Excessive inflammatory response was considered for a long time as a landmark of sepsis. However, sepsis is now recognized to involve early activation of both pro‐ and anti‐inflammatory responses.^1^ Therefore, the modulation of the inflammatory response in sepsis is an attractive target for therapy and the citrullination process opens a new research avenue for study.

As we mentioned above, the citrullination of histones, in particular histone H3, is a post‐translational modification that regulates gene expression and plays a critical role in NET development and in inflammatory and host defense response.^2^, ^3^, ^4^, ^5^, ^6^ This reaction is catalyzed by PAD4. The role of PAD4 in sepsis was only studied in experimental models of sepsis.^5^, ^7^, ^8^ Li et al^5^ showed that PAD inhibition significantly suppresses citrullinated histone H3 production in vitro, and improves survival in septic mice. In another study with PAD4 (PAD4^−/−^) deficient mice, the mice were partially protected from lipopolysaccharide‐induced shock, suggesting that PAD4/NETs may contribute to the toxic inflammatory and procoagulant host response to endotoxin.^7^ Recently, Biron et al^8^ also showed that Cl‐amidine treatment, an inhibitor of PAD4, prior to cecal ligation and puncture, improved overall survival in sepsis.

Thus, these studies suggest that PAD4 inhibition could improve sepsis mortality through the reduction of circulating citrullinated histone 3 and NET development. NETs are composed of DNA, histones and microbicidal peptides, with the ability to kill some bacterial species.^15^ The NETs immobilize and kill invading microorganisms to prevent their spreading. In addition to their host‐protective role during infection, the excessive formation of NETs is associated with tissue damage and organ dysfunction, in many pathological conditions, such as sepsis.^16^

Although circulating PAD4 could play a role in the pathogenesis of septic shock, PAD4 activity requires reducing conditions that could be meet only in the intracellular space. Thus, in this scenario, PAD4 could be interpreted as a marker of NETosis.^17^, ^18^

The noteworthy finding in the present study was that higher PAD4 concentrations were associated with mortality in septic shock patients. This finding confirms experimental data, and encourages further studies with PAD4 inhibition in humans, including the use of GSK199 and GSK484, the first inhibitors with a strong preference for PAD4.^19^ Importantly, PAD4 concentration remains associated with ICU mortality even when adjusted for age, gender and APACHE II score. It is worth mentioning that this is the first study to evaluate the role of *PADI4* polymorphisms in patients with septic shock.

The onset and progression of rheumatoid arthritis has been associated with dysregulated PAD activity, most prominently PAD4. Hashemi et al^20^ showed that *PADI4 rs1748033* gene polymorphism increased the risk of rheumatoid arthritis in a sample of the Iranian population. Two different meta‐analyses showed that *PADI4* polymorphisms, such as *PADI4*_89, *PADI4*_90, *PADI4_92*,* PADI4_94* and *PADI4*_104, were associated with rheumatoid arthritis in the Asian population.^10^, ^21^ Single nucleotide polymorphisms (SNPs) are the most common type of genetic variation, and some of them are thought to be functional. Our hypothesis was that individuals carrying the variant alleles could influence septic shock mortality. However, in this study, *PADI4* polymorphism genotypes and the haplotype block formed by *rs2240340* and rs*1748033* did not influence PAD4 concentration or mortality in patients with septic shock.

Some limitations of this study should be taken into account. We only included patients from a single center and our sample size was small. In addition, the only marker of inflammation that we measured was CRP. Nevertheless, we believe that our study added important data on inflammatory modulation pathways and its association with septic shock outcomes.

In conclusion, PAD4 concentration, but not its polymorphisms, is associated with ICU mortality in septic shock patients.

## CONFLICT OF INTEREST

The authors confirm that there are no conflicts of interest.

## AUTHOR'S CONTRIBUTIONS

PSA, NBC, BFB, ESM, LLWI, RCSB, EAS, RBG: acquisition of subjects and data, analysis and interpretation of data, revising the article critically; MMR, MMN: interpretation of genotype data and revising the article critically; SET: statistical analysis and revising the article critically; NAC, SARP, MFM, LAMZ, ALG: study design, analysis and interpretation of data, and drafting the manuscript. All authors revised the article critically for important intellectual content, and approved the final version of the manuscript.

## References

[1] Singer M , Deutschman CS , Seymour CW , et al. The third international consensus definition for sepsis and septic shock (Sepsis‐3). JAMA. 2016;315:801‐810. 26903338 10.1001/jama.2016.0287 PMC4968574

[2] Baka Z , Gyorgy B , Géher P , Buzás EI , Falus A , Nagy G . Citrullination under physiological and pathological conditions. Joint Bone Spine. 2012;79:431‐436. 22366145 10.1016/j.jbspin.2012.01.008

[3] Nguyen H , James EA . Immune recognition of citrullinated epitopes. Immunology. 2016;149:131‐138. 27531825 10.1111/imm.12640 PMC5011684

[4] Fuhrmann J , Clancy KW , Thompson PR . Chemical biology of protein arginine modifications in epigenetic regulation. Chem Rev. 2015;115:5413‐5461. 25970731 10.1021/acs.chemrev.5b00003 PMC4463550

[5] Li Y , Liu Z , Liu B , et al. Citrullinated histone H3: a novel target for the treatment of sepsis. Surgery. 2014;156:229‐234. 24957671 10.1016/j.surg.2014.04.009 PMC4267527

[6] Margraf S , Logters T , Reipen J , Altrichter J , Scholz M , Windolf J . Neutrophil‐derived circulating free DNA (cf‐DNA/NETs): a potential prognostic marker for posttraumatic development of inflammatory second hit and sepsis. Shock. 2008;30:352‐358. 18317404 10.1097/SHK.0b013e31816a6bb1

[7] Martinod K , Fuchs TA , Zitomersky NL , et al. PAD 4‐deficiency does not affect bacteremia in polymicrobial sepsis and ameliorates endotoxemic shock. Blood. 2015;125:1948‐1956. 25624317 10.1182/blood-2014-07-587709 PMC4366625

[8] Biron BM , Chung CS , O'Brien XM , Chen Y , Reichner JS , Ayala A . CI‐Amidine prevents histone 3 citrullination and neutrophil extracellular trap formation, and improves survival in a murine sepsis model. J Innate Immun. 2017;9:22‐32. 27622642 10.1159/000448808 PMC5219946

[9] Kobayashi T , Ito S , Kobayashi D , et al. Serum immunoglobulin G levels to porphyromonas gingivalis peptidylarginine deiminase affect clinical response to biological disease‐modifying antirheumatic drug in rheumatoid arthritis. PLoS ONE. 2016;11:e0154182. 27111223 10.1371/journal.pone.0154182 PMC4844134

[10] Lee YH , Bae SC . Association between susceptibility to rheumatoid arthritis and PADI4 polymorphisms: a meta‐analysis. Clin Rheumatol. 2016;35:961‐971. 26474773 10.1007/s10067-015-3098-4

[11] Dellinger RP , Levy MM , Rhodes A , et al. Surviving Sepsis Campaign: international guidelines for management of severe sepsis and septic shock: 2012. Crit Care Med. 2013;41:580‐637. 23353941 10.1097/CCM.0b013e31827e83af

[12] Maintinguer Norde M , Oki É , de Castro IA , et al. Influence of adiponectin gene variants and plasma fatty acids on systemic inflammation state association‐A cross‐sectional population‐based study, São Paulo, Brazil. Mol Nutr Food Res. 2016;60:278‐286. 26419856 10.1002/mnfr.201500527

[13] Costa NA , Gut AL , Pimentel JA , et al. Erythrocyte selenium concentration predicts intensive care unit and hospital mortality in patients with septic shock: a prospective observational study. Crit Care. 2014;18:R92. 24887198 10.1186/cc13860 PMC4057214

[14] Machado FR , Mazza BF . Improving mortality in sepsis: analysis of clinical trials. Shock. 2010;34:54‐58. 20523272 10.1097/SHK.0b013e3181e7e8b4

[15] Yang H , Biermann MH , Brauner JM , Liu Y , Zhao Y , Herrmann M . New insights into Neutrophil Extracellular Traps: mechanisms of formation and role in inflammation. Front Immunol. 2016;7:302. 27570525 10.3389/fimmu.2016.00302 PMC4981595

[16] Czaikoski PG , Mota JM , Nascimento DC , et al. Neutrophil extracellular traps induce organ damage during experimental and clinical sepsis. PLoS ONE. 2016;11:e0148142. 26849138 10.1371/journal.pone.0148142 PMC4743982

[17] Damgaard D , Bjorn ME , Steffensen MA , et al. Reduced glutathione as a physiological co‐activator in the activation of peptidylarginine deiminase. Arthritis Res Ther. 2017;18:102. 10.1186/s13075-016-1000-7 PMC4858833 27149996

[18] Damgaard D , Bjorn ME , Jensen PO , Nielsen CH . Reactive oxygen species inhibit catalytic activity of peptidylarginine deiminase. J Enzyme Inhib Med Chem. 2017;32:1203‐1208. 28933232 10.1080/14756366.2017.1368505 PMC6021033

[19] Lewis HD , Liddle J , Coote JE , et al. Inhibition of PAD4 activity is sufficient to disrupt mouse and human NET formation. Nat Chem Biol. 2015;11:189‐191. 25622091 10.1038/nchembio.1735 PMC4397581

[20] Hashemi M , Zakeri Z , Taheri H , Bahari G , Taheri M . Association between Peptidylarginine Deiminase Type 4 rs1748033 Polymorphism and Susceptibility to Rheumatoid Arthritis in Zahedan, Southeast Iran. Iran J Allergy Asthma Immunol. 2015;14:255‐260. 26546893

[21] Yang XK , Liu J , Liu J , et al. Associations between PADI4 gene polymorphisms and Rheumatoid Arthritis: an updated meta‐analysis. Arch Med Res. 2015;46:317‐325. 26043831 10.1016/j.arcmed.2015.05.011

